# Radicalization of Glyceraldehyde-3-Phosphate Dehydrogenase by HOCl in Living Cells

**Published:** 2015

**Authors:** Sandra E Gomez-Mejiba, Zili Zhai, Marcos D Muñoz, Maria C. Della Vedova, Kalina Ranguelova, Michael T Ashby, Dario C Ramirez

**Affiliations:** 1 Laboratory of Experimental Therapeutics-IMIBIO-SL-CONICET and School of Health Sciences, National University of San Luis, San Luis, San Luis 5700, Argentina; 2 Department of Dermatology, University of Colorado Denver, Aurora, 80045 CO, USA; 3 Laboratory of Experimental and Translational Medicine-IMIBIO-SL-CONICET and School of Chemistry, Biochemistry and Pharmacy, National University of San Luis, 5700 San Luis, San Luis, Argentina.; 4 Bruker BioSpin Corporation, Billerica, MA 01821, USA; 5 Department of Chemistry and Biochemistry, University of Oklahoma at Norman, OK 73019, USA

**Keywords:** GAPDH, Macrophage, Lipopolysaccharide, Reactive chemical species, Cell death, Protein radical, Immuno-spin trapping

## Abstract

A number of post-translational oxidative modifications of the enzyme “cell-redox sensor” glyceraldehyde-3-phosphate dehydrogenase (GAPDH) have been reported. These modifications affect GAPDH structure, function, and cell fate; however no free-radical mechanisms have been reported in these processes. Herein we used the nitrone 5,5-dimethyl-1-pyrroline *N*-oxide (DMPO)-based spin trapping techniques to examine a novel free radical mechanism that causes GAPDH inactivation and aggregation in RAW264.7 cells primed with lipopolysaccharide (LPS). In these primed cells, GAPDH is oxidized by myeloperoxidase (MPO)-derived hypochlorous acid (HOCl) resulting in loss of enzyme activity and aggregation, accumulation of lactate and cell death. Due to the close spatial and physical proximity between MPO and GAPDH, and the oxidizing potential of HOCl, it may be the main species that triggers radicalization of GAPDH that ultimately results in enzyme aggregation and inactivation in LPS-primed macrophages. Lysine residues are the primary radicalization sites formed upon reaction of HOCl with the enzyme. Our data highlight the important relationship between radicalization of GAPDH and fate of stressed cells, which might help teasing out the cell response to stress at sites of inflammation.

## Introduction

Glyceraldehyde-3-phosphate dehydrogenase (GAPDH**, EC 1.2.1.12) is a tetrameric enzyme (37 kDa per subunit) that uses NAD^+^ as electron acceptor to catalyze the oxidative phosphorylation of D-glyceraldehyde 3-phosphate to 1,3-biphosphoglycerate within the glycolytic pathway [1]. In recent years, a number of other direct and indirect functions of this protein have been discovered [1–3], including assisting nuclear membrane fusion, microtubule bundling, phosphotransferase activity, RNA export, prostate cancer progression, apoptosis, DNA replication and repair, and maintenance and/or protection of telomeres. All mammalian GAPDHs so far examined have an exceptionally reactive Cys^149^ residue that is essential for enzyme activity which modification affects its glycolytic activity as well as aggregation state [4]. The aggregation of GAPDH has been shown to be important in nuclear translocation and cell death [2–5].

A number of post-translational modifications in GAPDH by non-radical [6–8] and electrophilic mechanisms, [9,10] in particular on its Cys^149^ residue, have been reported to cause cell death. For instance, GAPDH is inhibited when it undergoes S-nitrosylation by ^•^NO, NAD^+^ covalent linkage on S-nitrosylation, nitration by nitrated fatty acids, and S-glutathionylation by glutathione or by ^•^NO, [11] as well as extensive oxidation disulfide (Cys^149^-Cys^153^) and protein aggregation by H_2_O_2_ [4–8]. H_2_O_2_ and other peroxides can cause reduced glutathione (GSH)-mediated S-thiolation of GAPDH in endothelial cells [10]. Peroxynitrite, a cytotoxic reactive nitrogen species generated from inducible nitric oxide synthase (iNOS) in macrophages activated with lipopolysaccharide (LPS), can nitrate GAPDH [12] Nitration on two tyrosine residues (Tyr^311^ and Tyr^317^) blocks the access and binding of the electron acceptor NAD^+^ and thus inhibits enzyme activity [13]. These oxidative changes affect not only the glycolytic function but also aggregation state and subcellular compartmentalization, and they promote the participation of GAPDH in cell death [3].

GAPDH oxidation and aggregation has been reported in a number of diseases in which macrophages and myeloperoxidase (MPO)-derived reactive species have been observed, including Alzheimer’s, Lou Gehrig’s, Huntington’s, and Parkinson’s diseases [2]. Although, MPO produces a number of halides and pseudohalides, this is the only mammalian enzyme that produces hypochlorous acid or hypochlorite (HOCl/OCl^−^, pKa=7.58) under relevant physiological conditions. The thiolate function at the Cys^149^ residue (pKa=8.2) in GAPDH is easily oxidized by HOCl causing irreversible enzyme inactivation [14]. Although it has been shown that oxidizing compounds produced by MPO, such as hypothiocyanous acid, [15] HOCl and derived-chloramines(14) oxidize the Cys^149^ residue at the active site of the enzyme and causes enzyme inactivation, to our knowledge no studies prior to the present one have demonstrated formation of a protein radical in the death process of stressed cells.

Macrophages treated with LPS increase their production of reactive chemical species (e.g.; ^•^NO, ^•^O_2_^−^, ONOO^−^ and HOCl), synthesis of inflammatory cytokines (e.g., TNF-α, IL-6, and IL-1β) and enzymes (iNOS, GAPDH and cyclooxigenase-2), activation of NADPH oxidase-2 (NOX-2), oxidative damage to macromolecules and cell death. From the interplay between NOX-2, iNOS and MPO a number of oxidants that damage macromolecules and deplete antioxidants can be generated in the macrophage stressed with LPS causing oxidative stress. Oxidative modification of proteins, in particular GAPDH in LPS-stressed macrophages may determine cell fate. The involvement of free radical processes during the inactivation and aggregation of GAPDH in cells might be possible, but has until now remained unproven because of the technological limitations associated observing protein radicals *in situ* and in real time within cells. MPO is one of the enzymes that produce reactive chemical species in LPS-activated RAW264.7 cell [16–18] and primary macrophages [19–21] MPO has been shown to be a nuclear antigen in myeloid cells, such as HL-60 cells, that bind to genomic DNA [22]. After hydroxyl radical, HOCl is one of the most reactive and oxidizing species known in biology. HOCl can oxidize proteins with formation of protein-centered radicals as transient intermediates, which decay leads to protein fragmentation and/or aggregation [23]. These changes in the structure of critical proteins such as GAPDH can cause cytotoxicity if the damage is not stopped, or the affected protein is repaired or removed [5]. Protein-[nitrogen, sulfur or carbon]-centered radicals can be trapped by the nitrone spin trap 5,5-dimethyl-1-pyrroline N-oxide (DMPO) to form nitrone adducts and thus prevent electron transfer, cross-links and fragmentation [24]. Protein-DMPO nitrone adducts can be measured and studied in cells and in proteins using immuno-spin trapping assays [25] and references cited therein).

Herein we have used DMPO-based electronspin resonance spectroscopy and immuno-spin trapping to investigate the free radical mechanisms of oxidation of GAPDH and resulting changes in its structure and function in macrophages primed with LPS. We also determined the critical role of the oxidants formed in the interplay between NOX-2, iNOS and MPO on GAPDH radical formation, loss of activity and aggregation of the enzyme, and lastly cell death. These results reveal a novel mechanism of GAPDH oxidation in stressed cells; this mechanism may impact our understanding of the pathogenesis of a number of chronic inflammatory conditions, such as cell response to endogenous and exogenous stressors in which oxidation of GAPDH is known to contribute to cell death.

## Experimental Procedures

### Reagents

The murine peritoneal macrophage-type cell line RAW 264.7 was acquired from the American Type Culture Collection (Cat. no. TIB-71, Rockville, MD). DMPO and rabbit polyclonal anti-DMPO antibody were purchased from Alexis Biochemicals (San Diego, CA). LPS (*Escherichia coli* serotype 055:B5, L2367), rabbit muscle GAPDH (G2267), rabbit polyclonal anti-iNOS antibody, highly purified human erythrocytic GAPDH, mouse anti-GAPDH antibody, mouse monoclonal anti-β-actin antibody, rabbit anti-nitrotyrosine, and sheep anti-mouse IgG Cy3 conjugate were from Sigma (St. Louis, MO). Rabbit anti-human MPO antibody was from Athens Research and Technology (Athens, GA). The monoclonal anti-mouse MPO antibody was from Cell Sciences (Canton, MA). The rabbit anti-p47^*phox*^ antiserum, goat anti-rabbit IgG, goat anti-mouse IgG conjugated with horseradish peroxidase (HRP) and protein A/G Plus-agarose immunoprecipitation reagent were from Santa Cruz Biotechnology (Santa Cruz, CA). The goat anti-rabbit DyLight 649 was from Thermo Fisher Scientific (Rockford, IL).

### Cell culture

RAW 264.7 cells were cultured in high-glucose Dulbecco’s Modified Eagle’s Medium (Sigma) supplemented with 5% fetal bovine serum, referred to as complete medium. The cells were maintained in a humidified atmosphere of 5% CO_2_ at 37°C and split 2–3 times weekly. Before experiments, newly split cells were allowed to adhere overnight. Then, the medium was removed and replaced by the indicated concentrations of LPS and/or DMPO in complete medium.

### Preparation of cell homogenates

For MPO extraction or for the determinations of MPO activity the homogenates were prepared as follow: After incubation, cells were washed twice with ice-cold phosphate buffered saline (PBS, 10 mM Na_2_HPO_4_, 1.8 mM KH_2_PO_4_, 2.7 mM KCl, 137 mM NaCl, pH=7.4) and then suspended and sonicated in 0.5% (w/v) hexadecyltrimethylammonium bromide-containing 10 mM sodium phosphate buffer (PB, 1.9 mM NaH_2_PO_4_ and 8.1 mM Na_2_HPO_4_), pH 6.0 [26] After a total of three cycles of freeze-thaw-sonication, the resultant supernatants were collected by centrifugation (11,700 × g for 15 min at 4°C). The whole cell homogenates were dialyzed overnight against 1 L of 10 mM PB containing 100 mM Cl^−^, pH 7.4. The dialyzed cell homogenates were stored at −80°C until use. For western blot analysis of other proteins, the cell homogenate was prepared as follow: Cell were washed and homogenized in RIPA lysis buffer containing 1% (v/v) protease inhibitor cocktail (Amresco Inc., Solon, OH) and 25 U/ml of Benzonase^™^ nuclease (Novagen, Madison, WI). The supernatants were collected by centrifugation (11,700 × g for 15 min at 4°C), and total protein concentration was determined using a BCA protein assay kit (Pierce Labs, Rockford, IL).

### Determination of GAPDH activity

The activity of GAPDH in macrophage homogenate was measured spectrophotometrically by monitoring the formation of NADH at 340 nm [27]. The reaction was performed by addition of cell homogenates (1 mg/ml) to a solution containing 50 mM PB, pH 8.9, 5 mM ethylenediaminetetraacetic acid (EDTA), 0.5 mM β-NAD^+^, and 0.5 mM glyceraldehyde-3-phosphate. The enzyme activity was calculated from the linear increase in absorbance between 0 and 1 min. One unit of GAPDH activity is defined as the amount of enzyme that catalyzes the formation of 1 μmol of NADH per min at pH 8.9 at 25°C.

### Determination of lactate

The lactate levels in cell culture medium were determined using a commercial kit following the manufacturer’ instructions (Cat. no K627, Biovision, Mountain View, CA).

### GADPH-DMPO nitrone adduct production in chemical systems

Purified rabbit muscle GAPDH was incubated with 2 mM 2-mercaptoethanol and 1 mM EDTA for 30 min at 4°C in the dark, and then dialyzed against 10 mM PB, pH 7.4, at 4°C overnight. The thiol concentration of the dialyzed GAPDH was determined by reduction of 5,5’-dithio-2-nitrobenzoic acid to 5-thio-2-nitrobenzoic acid at 410 nm using a molar extinction coefficient of 13.6 mM^−1^ cm^−1^ [14,28]. Typically, rabbit muscle GAPDH (10 μM as free thiols) was reacted with different oxidants, sources of oxidants or a MPO/H_2_O_2_/Cl^−^ system in 10 mM PB, pH 7.4, for 10–15 min, then DMPO was added to trap GAPDH-centered radicals. After 1 h of incubation, the reaction was stopped by adding 10 mM methionine or a mixture of 1 IU/ml catalase plus 10 mM diethylene triamine pentaacetic acid (DTPA). GAPDH-DMPO nitrone adducts in the reaction mixture were analyzed by enzyme-linked immuno-sorbent assay (ELISA) or Western blotting [25] with an anti-DMPO antibody [29].

### Chemical modification of cysteine, tyrosine, tryptophan, histidine, and lysine residues on GAPDH

Purified rabbit muscle GAPDH was used in this study. This protein (Protein Data Bank accession number 1J0X) contains 4 Cys, 7 Tyr, 3 Trp, 10 His, and 26 Lys residues per chain. Cysteine residues on GAPDH were blocked by alkylation with *N*-ethylmaleimide (NEM) at a NEM:Cys molar ratio of 20:1 for 30 min at room temperature in 0.1 M PBS, pH 7.4 with 100 μM DTPA [28]. Tyrosine residues were blocked by iodination using Iodo-Beads (Thermo Fisher Scientific) [30]. Tryptophan residues were blocked by incubating GAPDH with *N*-bromosuccinimide (NBS) in 0.1 M sodium acetate buffer, pH 4.5, at a NBS:Trp molar ratio of 2:1 for 15 min at room temperature. At the end of the incubation time, the reaction was neutralized with addition of 1 M NaOH. Histidine residues were blocked with diethylpyrocarbonate (DEPC) by the procedure of Miles [31]. Briefly, a solution of GAPDH was reacted with DEPC in 50 mM PBS, pH 6.0, at a DEPC:His ratio of 10:1 at room temperature for 60 min. The reaction was stopped by addition of a 10 molar excess of L-histidine. Lysine residues were blocked by succinylation using succinic anhydride (SA). GAPDH was incubated with SA in 0.2 M sodium borate buffer, pH 8.5, at a SA:Lys molar ratio of 5:1 for 30 min. SA was added in 4 steps to increase specificity of the modification to only lysine residues. In all cases, after the modification reactions the excess reagent was eliminated by dialysis using dialysis cassettes with a cut-off of 3.5 kDa against 2 L of 10 mM PBS, pH 7.4. The protein concentration was adjusted using the BCA protein assay kit (Thermo Fisher Scientific).

### MPO-driven protein-DMPO nitrone adduct production in cell homogenates

To assess the activity of MPO in homogenates of macrophages activated with LPS, we measured the HOCl-dependent formation of protein-centered radicals by trapping them with DMPO. Briefly, dialyzed cell homogenates were mixed with the indicated concentrations of H_2_O_2_, Cl^─^, MPO inhibitors or HOCl scavenger at 37°C for 30 min, then 50 mM DMPO was added and the reaction continued for another 1 h. The reaction was stopped by addition of 1 mM DTPA/1 mM methionine to scavenge chloramines and addition of catalase (1 IU) to consume excess H_2_O_2_. The protein-DMPO nitrone adducts produced in the reaction mixture were determined by ELISA with anti-DMPO antibody or further immunoprecipitated for DMPO nitrone adduct assay by Western blot.

### ELISA for Protein-DMPO nitrone adducts and GAPDH nitration

Protein-DMPO nitrone adducts and GAPDH nitration produced in chemical systems and/or cell homogenates were determined by ELISA following a procedure similar to that described in ref. [32]. Briefly, the reaction mixture was diluted 1:20 in 0.1 M bicarbonate buffer, pH 9.6. Two hundred μl of the dilution was added into each well of a 96-well white ELISA plate (Corning Incorporated, Corning, NY), and incubated at 37°C for 90 min. Following washing with PBS plus 0.05% Tween-20 (PBST) and blocking with 2.5% BSA in PBS at 37°C for 1 h, the plates were incubated for 1 h at 37^o^C with rabbit polyclonal antiDMPO (1:1,000) or anti-nitrotyrosine (1:1,000) serum in PBST. After washing, the immunocomplexes were detected using goat anti-rabbit IgG-HRP conjugate and VisiGlo Chemilu HRP substrate solution (Amresco), and the luminescence was read using a microplate reader (Infinite M200, Tecan, NC).

### Western blot analysis

Homogenates (1 mg/ml) or reaction mixtures were mixed with 4 × SDS NuPAGE sample loading buffer (Invitrogen Corporation, Carlsbad, CA) and 100 mM 2-mercaptoethanol. After heat denaturation, equal amounts of protein were separated on 4–12% NuPAGE Bis-Tris Gels (Invitrogen), followed by blotting onto a nitrocellulose membrane. After blocking with 1% non-fat milk or 1% BSA in PBS, the immunoblot was performed by incubation with a primary antibody in washing buffer (0.1% milk or 0.1% BSA in PBST) overnight at 4°C, followed by incubation with HRP-conjugated goat anti-rabbit or goat anti-mouse IgG secondary antibodies (1:1,000 dilution) for 1 h at 37°C. The immunocomplexes were visualized using SuperSignal West Pico Chemiluminescent HRP Substrate (Thermo Fisher Scientific), and recorded in an Alpha Innotech Imaging system (San Leandro, CA). As primary antibodies we used the following antibodies at the dilutions indicated in parentheses: rabbit polyclonal anti-DMPO (1:1,000), anti-p47^phox^ (1:500), anti-MPO (1:500), anti-iNOS (1:500), anti-GAPDH (1:10,000), and anti-β-actin (1:2,500).

### Immunocytochemistry of MPO and GAPDH in macrophages

Cells were grown on 12 mm #1 round cover glasses. After treatment and rinsing with pre-warmed sterile PBS, cells were fixed with 4% paraformaldehyde in PBS for 15 min at 37°C, and then permeabilized with 0.2% Triton X-100 at room temperature for 10 min, followed by blocking with 2% BSA in PBS at 37°C for 1 h. Fixed cells were incubated with monoclonal anti-mouse MPO (1:100) and rabbit monoclonal anti-GAPDH antibody (1:1,000) at 37°C for 1 h, and then goat anti-rabbit DyLight 649 (Thermo Fisher) and sheep anti-mouse IgG Cy3 antibodies at 37°C for 1 h. Finally, cells were mounted on cover glasses with Prolong Gold antifade reagent with 4’,6-diamidino-2-phenylindole (DAPI; Invitrogen), and the immunostaining examined by confocal imaging using a Leica SP2 MP Confocal Microscope with a 63 ×1.4 oil immersion objective. In order to avoid the fried egg effect, which might lead to misinterpretation of the data, we acquired planar images through the cell nucleus.

### Immunoprecipitation using the anti-DMPO molecular “catcher”

We prepared an anti-DMPO molecular catcher as previously described [32]. Briefly, cell homogenates or protein-DMPO nitrone adduct-containing reaction mixtures were incubated with the catchers overnight at 4°C. After incubation the immunocomplexes were separated with a magnet, washed three times with washing buffer and re-suspended in 50 μl of elution buffer (Pierce). After removal of the beads with the magnetic field, the solution of eluted proteins was mixed with 10 μl of sample buffer and 5 μl of 2-mercaptoethanol. After heating at 90°C for 10 min the proteins were assayed by Western blot for anti-DMPO and anti-GAPDH.

### Co-Immuno-precipitation experiments

To test the physical interaction between GAPDH and MPO, 60 μg/ ml GAPDH was incubated with or without 10 μg/ml MPO in Hanks buffered saline solution with Ca^2+^ and Mg^2+^ (HBSS^+^) for 1 h at 37°C. The incubation was performed in a Thermomixer at 200 rpm and with a total volume of 300 μl. alternatively, we loaded A549 epithelial cells with human MPO as previously described [18] followed by lysis of the cells in RIPA buffer and protein determination. MPO was pulled down from the protein mixture or cell homogenates with an anti-human MPO antibody or an anti-human GAPDH antibody and protein-A/G Plus agarose beads (Santa Cruz Biotechnology). The immunopurified proteins were separated in a reducing SDS-PAGE. After blotting the proteins onto a nitrocellulose membrane, a Western blot against GAPDH or MPO was performed.

### Electron spin resonance (ESR) spectroscopy

Samples were prepared by adding a final concentration of 200 mM DMPO to 1 mM GADPH and 5-fold or 75-fold molar excess over the protein of HOCl (added as a bolus) in a final volume of 250 μL. After initiation of the reaction by the addition of HOCl and briefly vortexed, the samples were transferred to a quartz ESR flat cell, and recording of the spectra was initiated within 1 min of the start of the reaction. ESR spectra were recorded using a Bruker E500 ESR spectrometer equipped with an ER4122SHQ microwave cavity and operating at 9.78 GHz and a modulation frequency of 100 kHz. The instrumental settings were as follows: field sweep, 80 G; microwave frequency, 9.78 GHz; microwave power, 20 mW, modulation amplitude, 1 G; conversion time, 82 ms; time constant, 82 ms; receiver gain, 5 × 10^4^; and number of scans, 4. Computer simulation was performed using WinSim software that is available to the public through the Internet (http://epr.niehs.nih.gov) [33].

### Statistical analysis

The quantitative data are reported as the mean values ± s.e.m from at least three experiments in duplicate. We determined the significance of differences between pairs by Student’s t-test and between treatments and controls by one-way ANOVA with Dunnett’s *post hoc* testing. The differences were considered to be statistically significant at P<0.05.

## Results

### LPS Exposure induces GAPDH inactivation, lactate accumulation and cytotoxicity in macrophages

**Figure 1:**
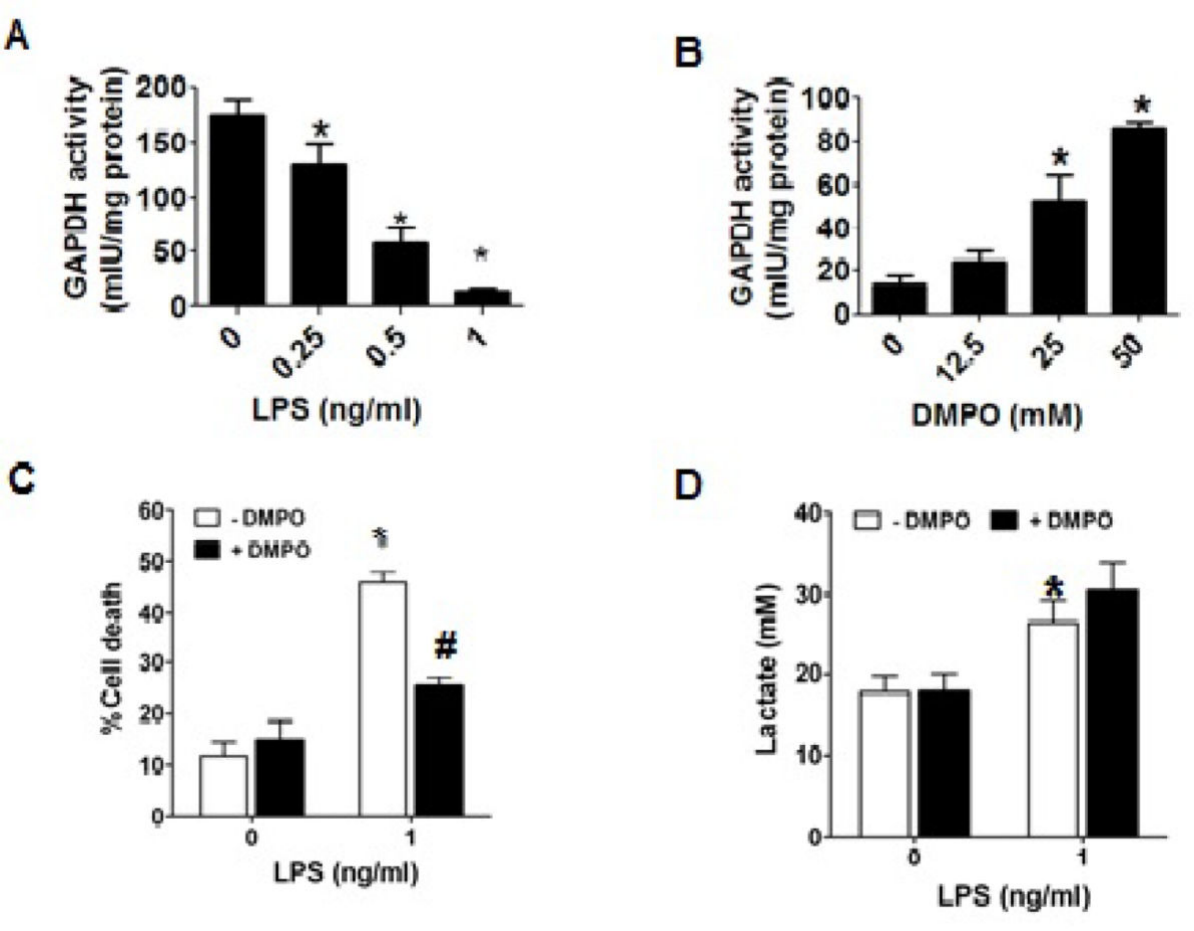
GAPDH activity, lactate production and viability in macrophages stressed with LPS. A) GAPDH activity in homogenate of RAW 264.7 cells treated with different concentrations of LPS for 24 h; B) as in A, but macrophages were treated with 1 ng/ml LPS and different concentrations of DMPO. C) Trypan blue exclusion assay in cells treated for 24 h with or without 1 ng/ml LPS and/or 50 mM DMPO. D) Determination of lactate in the supernatant of macrophages treated as in C. Data are expressed as mean values ± s.e.m. from three experiments run in duplicate. *p<0.05 vs. the corresponding untreated control; #p<0.05 vs. LPS-treated cells.

We found that upon 24 h incubation of macrophages with LPS the activity of GAPDH was inhibited in a concentration-dependent manner (Figure 1A). The nitrone spin trap DMPO that reacts with unpaired electrons in radicalized proteins can stop free radical-chain reactions, and thus protect against free radical-mediated oxidation and loss of biological function. The loss of GAPDH activity induced by LPS was partially prevented when DMPO was present into incubation medium (Figure 1B). However, with 50 mM DMPO, a concentration that does not cause significant cytotoxicity; only about 50% of the activity of the enzyme was protected. After 24 h incubation higher concentrations of DMPO caused cell death (data not shown). We next measured the effect of LPS and/or DMPO on cell viability using an assay based in the ability of the cell membrane to exclude Trypan blue dye. As observed in Figure 1C, exposure of macrophages to 1 ng/ml LPS for 24 h caused a 40–50% increase in cell death as compared with cells incubated in medium alone (10%). DMPO alone did not affect Trypan blue exclusion, however, and consistent with the effect on GAPDH activity, DMPO reduced LPS-induced cytotoxicity by almost 50% with respect to cells exposed to LPS alone (Figure 1C). Similar results were obtained measuring the release of lactic dehydrogenase (data not shown). To assess the metabolic effect of GAPDH inactivation produced by LPS, we measured the concentration of lactate in the culture medium of macrophages treated with or without 1 ng/ml LPS and/or 50 mM DMPO. Although we had expected that inactivation of GAPDH would reduce the production of lactate and the cell would die of starvation, we found that LPS increased lactate production by RAW264.7 cells (Figure 1D). One explanation to consider is that DMPO might increase lactate production because it protects GAPDH against inactivation; however, Figure 1D shows that DMPO does not change lactate production as compared to LPS alone. These findings suggest that upon oxidation of GAPDH, and maybe through other mechanisms as well, the cell may increase GAPDH synthesis as reported by Ravasi, [17] but the ratio of biologically active GAPDH to total GAPDH protein may decrease.

### Time-dependent changes in the activity of GAPDH and sources of oxidants

**Figure 2:**
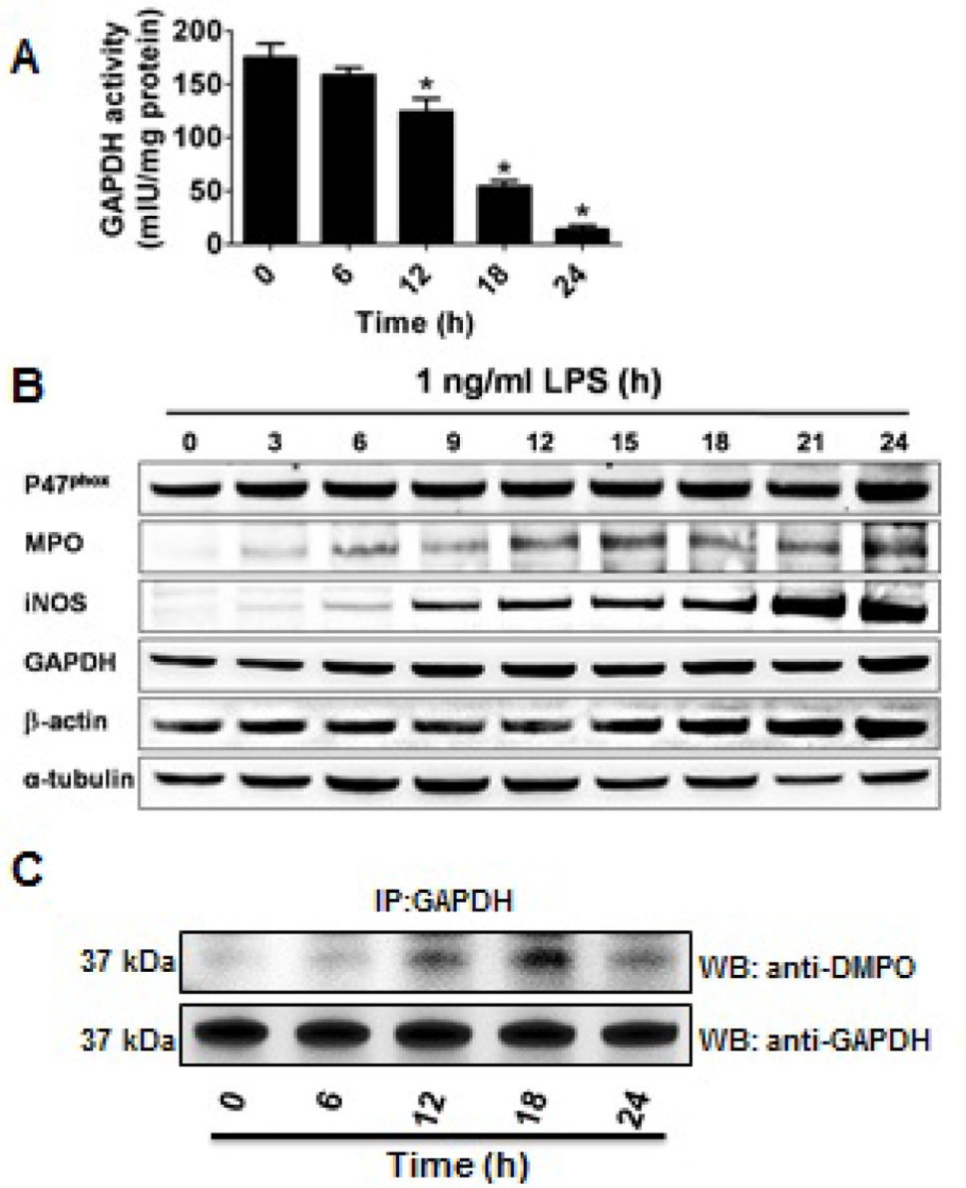
Time-dependent changes in GAPDH activity, source of oxidants and GAPDH-centered radicals incellstreatedwithLPS. A) GAPDH activity in homogenate of macrophages treated with 1 ng/ml LPS for the indicated time periods; B) Same as A, but shows the Western blot against p47phox, myeloperoxidase (MPO), iNOS and GAPDH protein content; C) Same as A, but the incubation contained 50 mM DMPO added with 1 ng/ml LPS at the beginning of the incubation. After incubation GAPDH was immunoprecipitated (IP) from the homogenates, separated by SDS-PAGE and blotted onto a nitrocellulose membrane. The blot was tested against DMPO and after stripping it was tested against GAPDH. Data shown are mean values ± s.e.m. from 3 independent experiments.

Oxidation of GAPDH during LPS-induced activation of macrophages may undergo time-dependent changes in the source, compartmentalization and nature of the reactive species formed. Consequently, we followed the activity of GAPDH in homogenates of macrophages exposed for different times to 1 ng/ml of LPS. Figure 2A shows that the activity of the enzyme starts decaying at about 12 h to reach 10% of the initial activity after 24 h of incubation. To assess the source of oxidants that may cause GAPDH-centered radicals that could lead to aggregation and inactivation of GAPDH, we then studied the expression of p47^*phox*^ (a subunit of NOX-2), MPO and iNOS, which are induced in macrophages treated with LPS [32] Remarkably, we found that between 6 and 12 h of incubation, the time range during which GAPDH activity starts decreasing, the amounts of NOX-2, iNOS, MPO and GAPDH protein increased suggesting that the interplay between oxidants generated from these sources might play a role in GAPDH inactivation (Figure 2B). We next immunoprecipitated GAPDH from homogenates of macrophages, treated with LPS and DMPO for different times, and then assayed for GAPDH and GAPDH-DMPO nitrone adducts. As shown in Figure 2C, the greatest amount of nitrone adduct was found at 18 h.

### Oxidants produced during activation of macrophages cause GAPDH-centered radicals

**Figure 3:**
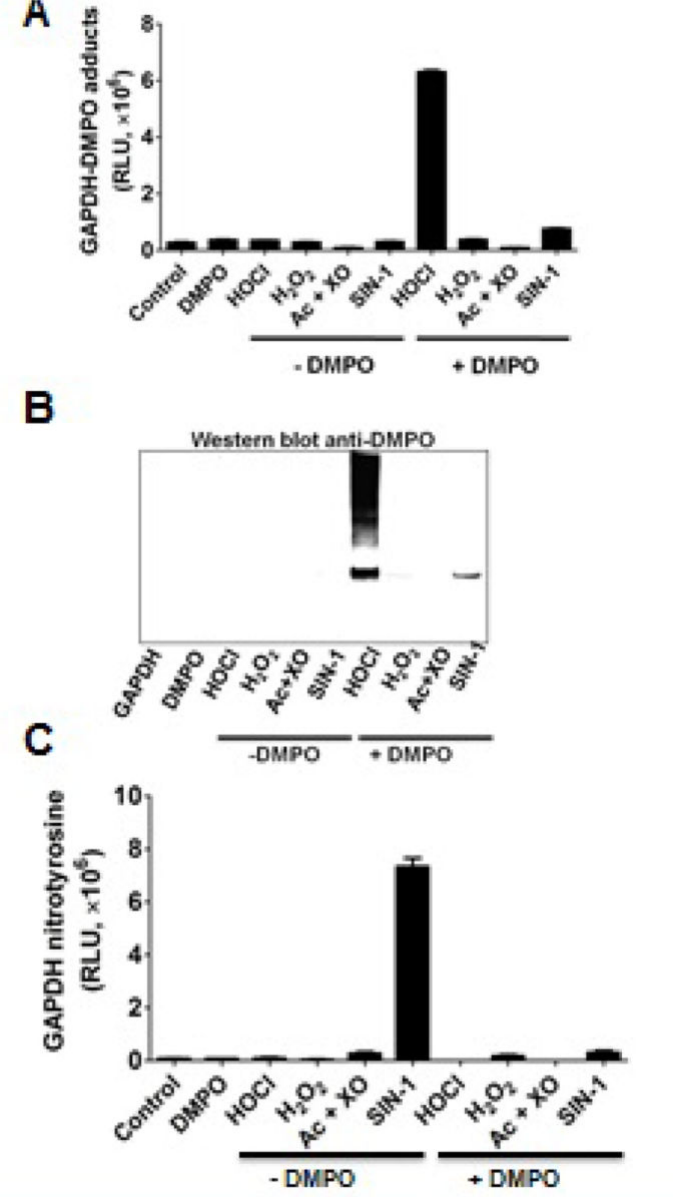
Effect of various oxidants produced during macrophage activation on GAPDH oxidation. A) The incubation mixture contained 10 μM (as free thiol) rabbit muscle GAPDH in Chelex-treated 10 mM sodium phosphate buffer, pH 7.4. The reaction was started by adding either 50 μM HOCl, 100 μM H_2_O_2_, 1 mM acetaldehyde (Ac)/ 30 mIU/ml xanthine oxidase (XO)—a source of superoxide, or 100 μM 3-morpholinosydnonimine (SIN-1)—a source of peroxynitrite. Incubations were performed in the absence or in the presence of 50 mM DMPO, which was added 15 min after starting the reaction, and the incubation was continued for 1 h more at 37°C. Reaction was stopped by dialysis against 1,000 volumes of phosphate buffer at pH 7.4. **B**) Western blot analysis of GAPDH nitrone adducts prepared as in A. **C**) Western blot anti-nitrotyrosine of the reaction mixtures prepared as in A. Incubations were performed in the absence or in the presence of 50 mM DMPO, which was added 15 min after starting the reaction, and the incubation was continued for 1 h more at 37°C. Reaction was stopped by dialysis at 4°C against 1,000 volumes of phosphate buffer. Data show a representative image from two independent experiments. Data are mean values ± s.e.m. or representative image from at least three independent experiments. RLU indicates relative light units.

^•^O_2_^−^ and ^•^NO are two of the most studied reactive species in cell signaling triggered by LPS in macrophages; however, by themselves they cannot form protein-centered radicals [32]. To avoid the complexity of the viscosity of the cytosol, multiple targets and reactive species scavenging systems in the cell, we used the well known biochemical model of GAPDH purified from rabbit muscle. We treated rabbit muscle GAPDH with a number of oxidants that are known to be formed in macrophages activated with LPS. Based on the time-dependent increase in NOX-2, iNOS and MPO proteins in macrophages treated with LPS (Figure 2B), we tested ^•^O_2_^−^, H_2_O_2_, HOCl and ONOO^−^ (peroxynitrite) to see whether they can form GAPDH-centered radicals that are trapped with DMPO (Figure 3A). Of the four reactive species tested, HOCl and ONOO^−^ (SIN-1) were the only reactive species that caused GAPDH-centered radicals, with HOCl substantially much more active than ONOO^−^ (Figure 3A). HOCl produced a marked amount of nitrone adduct and also extensive aggregation of the protein while ONOO^−^ produced much less of either (Figure 3B). Moreover, peroxynitrite, which is formed by the NOX2/iNOS system in LPS-activated RAW264.7 cells, [34] has also been involved in nitration and inactivation of GAPDH [12]. Nitration of GAPDH requires formation of a tyrosyl radical, which is then trapped by ^•^NO_2_ to form 3-nitrotyrosine [35]. Although as noted above ONOO^−^ produced a small amount of GAPDH-centered radical (Figure 3A), it did not produce visible aggregation (Figure 3B). We next competed nitration with nitrone adduct formation and observed that DMPO can block ONOO^−^-induced GAPDH nitration (Figure 3C).

### Myeloperoxidase produces oxidants that cause GAPDHcentered radical formation in LPS-activated macrophages

**Figure 4:**
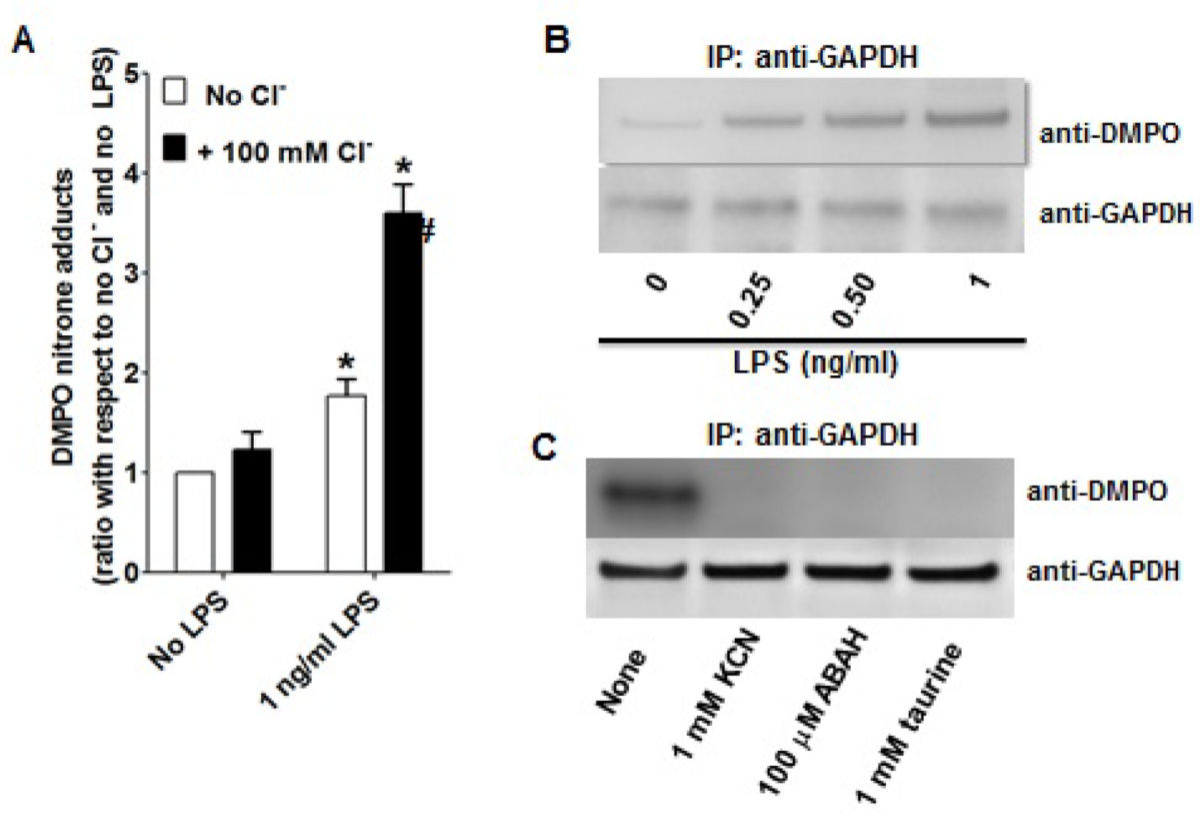
Active MPO in LPS-stressed RAW264.7 cells causes GAPDH-centered radicals. A) ELISA of total nitrone adducts produced in homogenate of macrophages treated or not treated with 1 ng/ml LPS for 24 h. The reaction mixture contained 1 mg/ml of dialyzed homogenate in 10 mM sodium phosphate buffer, pH 7.4, containing or not 100 mM chloride (as sodium salt). The generation of HOCl was started by addition of 100 μM H_2_O_2_; after 30 min of incubation 10 mM DMPO was added and the incubation continued for 1 h more. The reaction was stopped with methionine to scavenge excess HOCl and chloramines. *p<0.05 *vs* no LPS cells; #p<0.05 *vs.* the LPS-treated cells without Cl^−^; **B)** Dialyzed homogenates of macrophages treated with different concentrations of LPS for 24 h were then treated as in A with 100 μM H_2_O_2_ in the presence of chloride and 100 mM DMPO. After incubation, GAPDH was immunoprecipitated (IP) and separated by SDS-PAGE, blotted onto a membrane and tested by Western blot against DMPO. After stripping, the membrane was tested against GAPDH; **C)** As in A, but cyanide, ABAH or taurine was added before adding H_2_O_2_. IP of GAPDH and anti-DMPO/GAPDH Western blot analysis were performed as in B. Data are mean values ± s.e.m. (n=3) or a representative image from 3 independent experiments.

**Figure 5:**
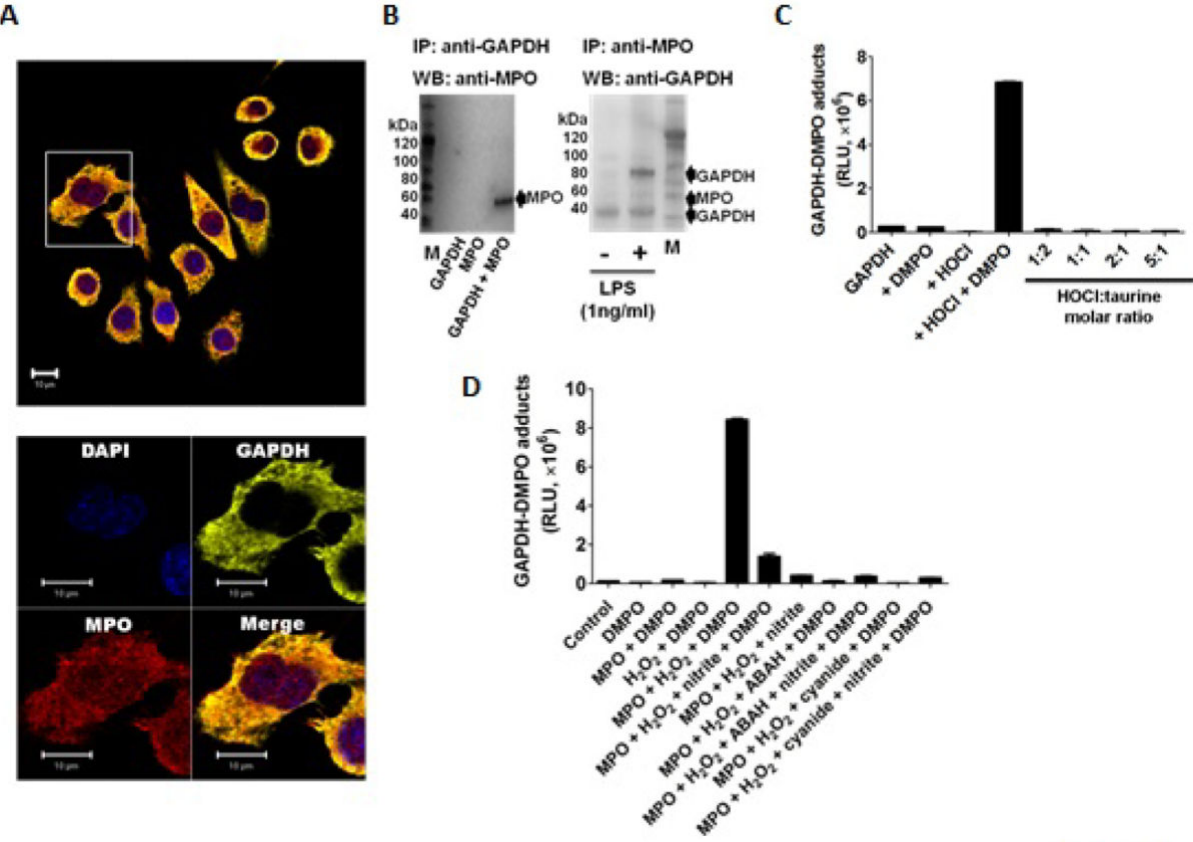
GAPDH physically interact with MPO in the same compartment in RAW264.7 cells stressed with LPS. **A)** Representative Single-plane confocal image showing intracellular distribution of GAPDH and MPO in macrophages treated with 1 ng/ml for 24 h. Scale bar is 10 μm. Lower panel shows a magnified image from the square marked in the top panel. Blue are the nuclei stained with DAPI; GAPDH is stained in green and MPO is stained in red. Observe punctuate pattern of MPO. **B**) *Left*, rabbit muscle GAPDH (60 μg/ml) was incubated for 30 min at 37°C with MPO (10 μg/ml) in Hank’s balanced saline solution with calcium and magnesium (HBSS^+^). GAPDH was immunoprecipitated (IP) from the reaction mixture, separated by SDS-PAGE and then were blotted onto a nitrocellulose membrane and tested against MPO; *Right,* RAW264.7 cells were treated with or without 1ng/ml of LPS for 24 h, followed by homogenization and IP with an anti-MPO antibody followed by an SDS-PAGE separation and Western blot anti-human GAPDH. M indicates MagicMark. **C)** Purified human MPO (10 nM) and rabbit muscle GAPDH (10 μM as free thiols) were incubated in 10 mM phosphate buffer, pH 7.4, with or without 100 mM chloride, 100 μM H_2_O_2_, 100 μM KCN, 100 μM ABAH or 1 mM nitrite. KCN, ABAH or nitrite were added immediately before H_2_O_2_, whereas 50 mM DMPO was added 15 minutes after addition of H_2_O_2_. After 1 h incubation at 37 °C the reaction was terminated with a methionine (10 mM)/catalase (1 IU/ml) mixture. **D)** ELISA of GAPDH-nitrone adducts produced by HOCl and taurine chloramines. Rabbit muscle GAPDH (10 uM) was treated with or without HOCl in the presence or absence of DMPO; or GAPDH was added to a mixture of 50 uM HOCl and taurine at different molar ratios in sodium phosphate buffer, pH 7.4. In both models, 50 mM DMPO was added 15 minutes after GAPDH addition and the reaction was continued for 1 h more. The reactions were stopped with 10 mM methionine. Other set of experiments Total nitrone adducts were determined by ELISA. Data are mean values ± s.e.m. (n=6) or a representative image from 3 independent experiments. RLU indicates relative light units.

To test whether MPO found in LPS-activated macrophages is enzymatically active, we used dialyzed homogenates of macrophages treated with or without 1 ng/ml of LPS for 24 h, and subsequently triggered HOCl formation by adding H_2_O_2_ in a buffer containing 100 mM chloride anion at pH 7.4. ELISA analysis of these experiments showed the highest production of DMPO-protein nitrone adducts when homogenates of macrophages primed with LPS was used (Figure 4A). Furthermore, to assess whether GAPDH is one of the DMPO-labeled proteins, we immunoprecipitated GAPDH from these homogenates and assessed nitrone adduct formation. Indeed, when HOCl was produced in homogenates of macrophages treated with LPS, there was a concentration-dependent increase in the amount of immunoprecipitated GAPDH-DMPO nitrone adducts (Figure 4B). Cyanide—a non-specific inhibitor of peroxidases, ABAH—a fairly specific inhibitor of MPO, and taurine—a scavenger of HOCl, blocked GAPDH nitrone adduct production when homogenates of macrophages treated with 1 ng/ml LPS for 24 h were treated with H_2_O_2_ (Figure 4C). Data shown in Figure 4C suggest that oxidants derived from HOCl [35,36] or HOCl itself might cause GAPDH-centered radicals in LPS-primed macrophages. We next addressed the factors that determine the specificity of GAPDH oxidation by MPO in LPS-primed macrophages. We rationalized that this specificity may be due to proximity whereby GAPDH is located in the same compartment as MPO in the LPS-activated macrophage. Indeed, confocal imaging showed that some MPO and GAPDH co-localize in LPS-primed macrophages (Figure 5A). Additionally, because of the cationic and highly glycosylated properties of MPO (PI>11), [36] we sought to test whether MPO physically interacts with GAPDH. To test this possibility we mixed rabbit muscle GAPDH with human MPO in a saline buffer followed by immunoprecipitation of GAPDH. We found that MPO was one of the proteins pulled down with the anti-GAPDH antibody (Figure 5B, left panel). Next we immunoprecipited MPO from homogenates of macrophages treated for 24 h with or without 1 ng/ml LPS, separated the proteins by SDS-PAGE, and tested against GAPDH by Western blot. We observed more GAPDH pulled down from homogenates of those cells treated with LPS (Figure 5B, right panel) suggesting that MPO and GAPDH physically interact in RAW264.7 cells treated with LPS.

This spatial and physical interaction does not necessarily demonstrate why GAPDH-centered radicals are formed in LPS-stressed cells. Therefore, we next sought to determine which oxidant species produced by the MPO-Cl^−^-H_2_O_2_ system can cause GAPDH-centered radicals. To accomplish this we incubated rabbit muscle GAPDH with highly purified human MPO in a buffer containing chloride with various mixtures of inhibitors and nitrite. Nitrite is one of the main ^•^NO oxidation products that accumulate during LPS activation in macrophages [37] (Figure 5C). We found that at 100 mM chloride, the addition of 1 mM nitrite decreased nitrone adducts by almost a factor of 6, suggesting that either nitrite competes more efficiently than chloride for compound I of MPO or, more likely, it reacts quickly with DMPO in solution. As expected, ABAH and cyanide inhibited MPOCl^−^-H_2_O_2_-induced GAPDH-nitrone adduct formation (Figure 5C) and protein aggregation (data not shown). We found that although taurine chloramine can inhibit GAPDH activity (data not shown, see also [14], it does not form GAPDH-centered radicals (Figure 5D).

### HOCl triggers a protein radical in GAPDH that is trapped by DMPO

**Figure 6:**
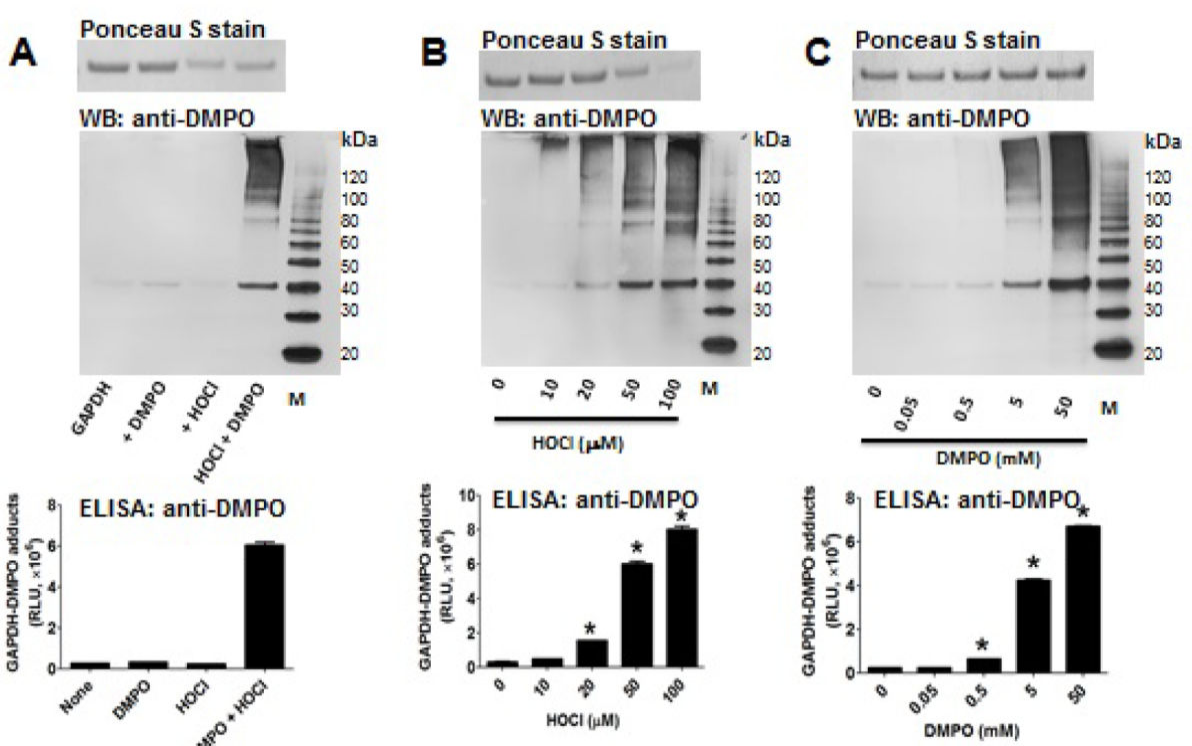
HOCl causes GAPDH-centered radicals as monomers and aggregates. In A-C, the *top panels* show a Ponceau S staining of the protein blotted onto a nitrocellulose membrane, *middle panels* show an anti-DMPO Western blot, and the *lower panels* show an anti-DMPO ELISA. M indicates molecular weight marker, and RLU indicates relative light units. **A)** Rabbit muscle GAPDH (10 μM as free thiol) was incubated with or without 10 μM HOCl and/or 10 mM DMPO. **B)** Same as in A, but GAPDH was incubated with different concentrations of HOCl followed, 10 min later, by addition of 10 mM DMPO; **C)** as in B, but GAPDH was incubated with 50 μM HOCl and different concentrations of DMPO were added 15 min later. *p<0.05 vs. the corresponding control (*i.e.,* no HOCl in B; no DMPO in. C). The reaction mixture was performed in 100 mM sodium phosphate buffer, pH 7.4, and the incubation was at 37°C for 1 h. The reaction was stopped with 10 mM methionine. Data show a representative image or mean values ± s.e.m. from 3 independent experiments in duplicate.

To test the ability of HOCl to cause GAPDH-centered radicals, we incubated rabbit muscle GAPDH with a bolus addition of a 5-fold excess of HOCl in the presence of DMPO. We found that HOCl produced GAPDH-nitrone adducts and that omission of any of the components in the reaction mixture resulted in no nitrone adduct formation (Figure 6A). In addition, HOCl-induced GAPDH aggregation was only partially inhibited by 10 mM DMPO (Figure 6A and 6B, top panels). Figure 6B, top and middle panels, show a HOCl-dependent aggregation of the enzyme as suggested by the decreased amount of its monomer form. We observed no fragmentation of GAPDH at the concentration of HOCl used in these experiments. Importantly, aggregates and monomers are found as their nitrone adducts (Figure 6A–C middle panel). Figure 6C (top) shows clearly the inhibitory effect of higher concentrations of DMPO on aggregation and the better yield of the nitrone adducts.

### Critical role of lysine residues as primary sites of radical formation in GAPDH treated with HOCl

**Figure 7:**
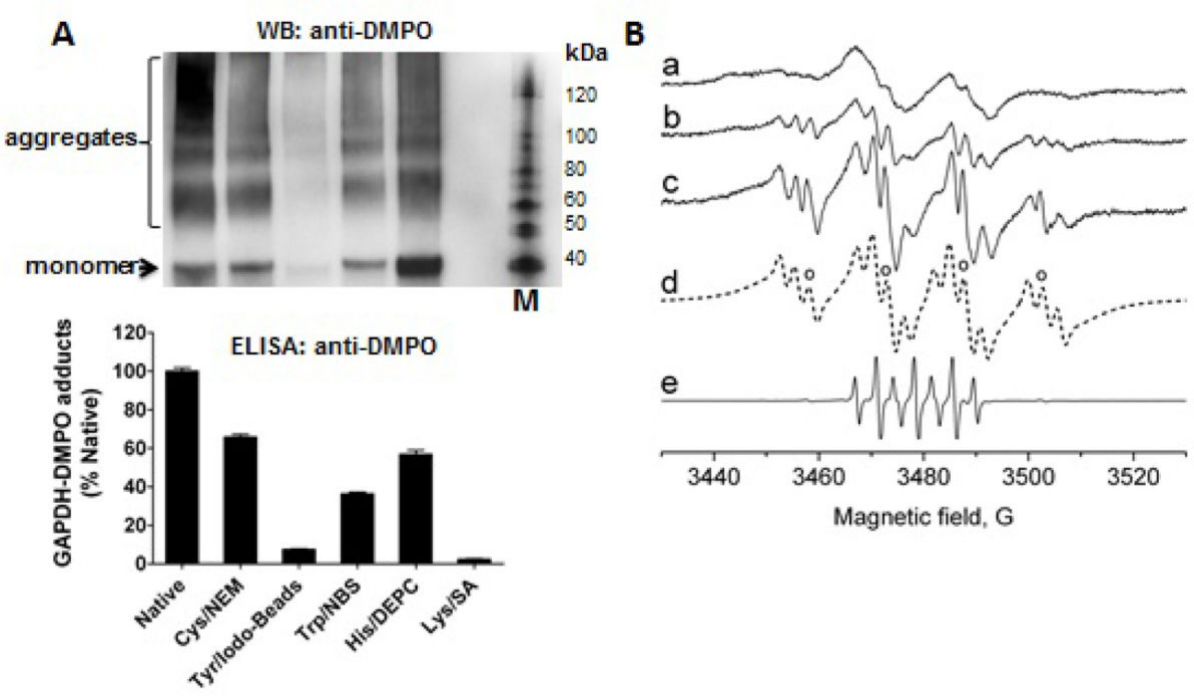
Effect of chemical modification of residues targets of radical attack on HOCl-induced GAPDH-centered radical formation. **A)** Western blot (*top*) and ELISA (*bottom*) of GAPDH-DMPO nitrone adducts. The reaction mixtures were prepared by reacting 10 μM native or chemically modified GAPDH with a 5 molar excess of HOCl at 37°C for 30 min in the presence of 50 mM DMPO. Data show a representative image or mean ± s.e.m. (n=4) from two independent experiments. M indicates the Magic Mark molecular weight marker. RLU indicates relative light units. NEM, *N*-ethylmaleimide—modifies cysteines; NBS, *N*-bromosuccinimide—modifies tryptophans; DEPC, diethylpyrocarbonate—modifies histidines; SA, succinic anhydride-modifies lysines. **B)** ESR spin trapping spectra of GAPDHcentered radicals trapped with DMPO. *Spectrum a*: the complete system (GAPDH (1 mM), and DMPO (200 mM) in 100 mM phosphate buffer, pH 7.4). After initiation with HOCl (5 mM), the mixture was immediately placed into the flat cell. *Spectrum b*: Pronase (0.2 mg/ml) was added to the sample that resulted in (spectrum a), and the solution was incubated for 5 min at room temperature before the spectrum was recorded. *Spectrum c*: The same sample as in (spectrum a) except the molar ratio HOCl:GAPDH was 75:1. *Spectrum d*: Composite computer simulation of (spectrum c) of the DMPO/^●^Lys radical adduct component (molar ratio 0.91) and DMPO/^●^OH radical adduct (molar ratio 0.09, marked with circles). *Spectrum e*: The same as in (spectrum c) except GAPDH was not added.

To determine which amino acid residue(s) is/are radicalized and trapped by DMPO in GAPDH, we chemically-modified amino acid residues that are usually sites of radical formation in proteins treated with HOCl [23]: cysteine, tyrosine, tryptophan, histidine and lysine residues; in rabbit muscle GAPDH. We then reacted the native and modified protein with a 5-fold excess of HOCl added as a bolus. Data shown in Figure 7A (top and bottom), suggest a critical role of exposed lysine and tyrosine residues as starting points in the free radical-chain reactions that are initiated when GAPDH is exposed to HOCl. It is possible that radicals are first located at a lysine residue and then transferred to tyrosine, tryptophan, or other residues, which is a pathway that is known to cause aggregation of proteins [23]. The reaction of cysteine with HOCl can form sulfenyl chloride, which can decompose to form a thiyl radical. Cysteine modification reduced the GAPDH nitrone adduct formation by approximately 40% with respect to the native enzyme (Figure 7A, bottom). Histidine modification reduced radical formation by almost 50%, suggesting that this residue might be a secondary target of radical formation and trapped by DMPO.

To corroborate immuno-spin trapping data and to identify a primary site of radical formation in GAPDH upon reaction with HOCl, we performed an ESRspin trapping experiment. Incubations of rabbit muscle GAPDH (1 mM) with HOCl (5 mM) in the presence of DMPO (200 mM) resulted in a broad ESR spectrum of a protein-derived immobilized radical adduct with partially resolved hyperfine couplings (Figure 7B, spectrum a). Treatment of the radical adduct with pronase resulted in the conversion of the immobilized nitroxide spectrum to an isotropic, freely rotating nitroxide (Figure 7B, spectrum b). The ESR lines observed after pronase (0.2 mg/ml) treatment exhibited hyperfine structure characteristic for a nitrogen-centered DMPO radical adduct. Higher intensity and resolution of the spectrum were achieved when the experiment was repeated using higher HOCl:protein ratio (75 : 1) (Figure 7B, spectrum c). The resolution of the hyperfine couplings allowed us to simulate the DMPO radical adducts (Figure 7B, spectrum d). Based on the simulation parameters two radical species were identified. The first one was assigned to N-centered ^●^Lys radical based on the hyperfine splitting (*a*^N^=14.80 G, *a*^H^=17.98 G, and *a*^N^=2.98 G) which was consistent with previous reports [38]. The second species was assigned to DMPO/^●^OH radical adduct (*a*^N^=14.93 G, *a*^H^=14.80 G, marked with circles). In the absence of GAPDH, a signal of 5,5-dimethyl2-pyrrolidone-*N*-oxyl (DMPOX) was detected (Figure 7B, spectrum e) due to the reaction of HOCl with the spin trap as described previously [39].

## Discussion

Herein we demonstrate radicalization of GAPDH in LPS-primed RAW264.7 cells. Our immuno-localization and co-immunoprecipitation experiments show that GAPDH and MPO co-localize and physically interact in LPS-primed macrophages. Our data suggest that HOCl, instead of chloramines, or other NOX-2/iNOS/MPO-derived oxidants causes GAPDH radicalization. MPO can produce a number of oxidants that cause protein nitration, carbonylation and chlorination [23,40,41]. Although by different mechanisms, hypothiocyanate (OSCN^−^), HOCl and taurinechloramine have been shown to inhibit GAPDH activity, [14] but evidence of a transient GAPDH-centered radical has not been provided. These new insights complement the extensive information available regarding GAPDH aggregation and modifications by non-radical and electrophilic post-translational mechanisms that are involved in cell death during the pathogenesis of neurodegenerative diseases [3–7].

GAPDH is a ubiquitous enzyme, however maybe other reasons explaining its preferred tendency forming a protein-centered radical in LPS-activated macrophages. LPS triggers the activation of NOX-2, and induces the expression of iNOS and MPO in macrophages. From the interplay between these enzymes a number of oxidants can be formed. They include superoxide radical anion, ^•^NO, ONOO^−^, H_2_O_2_, HOCl, NO_2_Cl, ^•^Tyr, and ^•^NO_2_ [42]. In this report we found that HOCl and ONOO^−^ oxidize GAPDH with formation of a transient protein radical, with HOCl>>>ONOO^−^.

HOCl produced in close proximity to GAPDH reacts with exposed Lys residues to form chloramines, which further decompose to form a nitrogen-centered radical. This radical can be translocated to other residues, for example Cys, Tyr, Trp and His. In addition, other one-electron oxidants such as ^•^NO_2_, NO_2_Cl or Tyr can directly produce Cys, Tyr, Trp or His radicals. ^•^NO_2_ and ^•^Tyr may be trapped by DMPO forming nitrone adducts. Protein-chloramines further decompose to form nitrogen-centered radicals that can then be transferred to carbon centers, including α-carbons, or to the amide nitrogen, leading to protein fragmentation and/or aggregation. Indeed, if not trapped by DMPO, protein-centered radicals decay by different mechanisms to form oxidation products such as disulfide-cross links, nitrated proteins and 2-mercaptoethanol-resistant aggregates, [23] which have been reported to play an important role in the toxic effect of oxidized GAPDH and cell death [5]. The cell’s fate under stressing conditions may depend on whether the modified protein can be repaired or degraded [43]. Our data show that 24 h after incubation, the amount of GAPDH nitrone adducts decrease compared to the amount observed at 18 h, suggesting that GAPDH tagged by DMPO may have been degraded and removed (Figure 2C). Our data also indicate that LPS induces and DMPO decreases cell death with formation of DMPO-nitrone adducts, suggesting that this effect afforded by DMPO might be because of its ability to trap protein-centered radicals before they convert to toxic inhibited enzymes or aggregates [4–5].

It is known that HOCl and taurine-chloramine inhibits GAPDH activity. However, ee did not observe GAPDH radicalization by taurine-chloramine, suggesting that it might act as a two-electron oxidant on cysteine residues critical for enzyme activity. In addition, our data show the critical role of lysine residues as starting points for HOCl-induced GAPDH radicalization. Although lysine and cysteine residues are important in the HOCl-initiated free radical process [23], other residues, such as tyrosine and tryptophan, might be secondary sites of radical localization in the protein. Likewise, previous work on the oxidation of bovine serum albumin by HOCl showed that the terminal side-chain amino groups of lysine residues are major targets for HOCl [38]. HOCl can oxidize several amino acid residues in proteins including Met>Cys>>Cysteine~His~α-amino>Trp> Lys>>Tyr ~Arg>Gln~Asn [44]. Our chemical modification and ESR spin trapping data are consistent with lysine residue(s) being the starting point(s) of the free radical chain process triggered by HOCl upon reaction with GAPDH (Figure 7). Interestingly, most of the aggregates generated upon oxidation of GAPDH with HOCl are resistant to 2-mercaptoethanol, suggesting the presence of other than disulfide cross-links in these aggregates. Previously it has been shown that nitrogen-centered radicals located at the ε-amino groups of lysine can form Schiff-base cross links with arginine residues, [23] which may result in 2-mercaptoethanol resistant aggregates. Moreover, GAPDH monomers and aggregates which have been shown in stressed cells, [4] are found in our model as nitrone adducts suggesting that more than one radical site is formed in the enzyme, and that some of them are trapped by the spin trap, whereas others are not. The aggregation also suggests that other radical sites may react faster to form cross-links than they do with DMPO [28]. Noteworthy is that other cross-links may be involved, but their formation may not necessarily involve protein-radical formation as intermediates.

Of particular interest is the protection afforded by DMPO against HOCl-induced inactivation and aggregation of GAPDH. This observation indicates that DMPO traps *in situ* radicals that are located on other residues from which electron-transfer to the Cys^149^ might be possible, thereby resulting in enzyme inactivation. Although our data have not demonstrated the formation of a cysteinyl radical, it is possible that it is formed and trapped by DMPO. HOCl-induced formation of protein radicals starts with a rapid reaction of sulfhydryl and amino functions in proteins with HOCl to form sulfenyl chloride or chloramines, [38] respectively. Sulfenyl chloride can decompose to form a thiyl radical [23] which can be trapped by DMPO [24]. Our competition assay between nitration and nitrone adduct formation suggest that ONOO^−^ causes formation of a tyrosyl radical in GAPDH for which DMPO and ^•^NO_2_ compete (Figure 3C). To our best understanding this is the first report of a tyrosyl radical in GAPDH that might be involved in enzyme aggregation and/or inactivation in stressed cell [5].

Finally, we have used DMPO-based immuno-spin trapping and ESR spin trapping to dissect a novel free radical mechanism of HOCl-induced GAPDH oxidation, inactivation, and aggregation in LPS-stressed macrophages-like cells. MPO is the major source of oxidants that cause GAPDH oxidation inside LPS-stressed macrophages. This is the first report of HOCl-induced GAPDH-centered radicals in functioning and stressed cells. This oxidation is triggered by the reaction of HOCl, mostly with exposed lysine residues, from where an electron can be transferred to cysteine residues critical for GAPDH activity. The information provided herein will complement the previous studies of non-free radical-mediated modification of the function and structure of GAPDH and cell death. The present findings, together with our recently published findings of uptake of MPO and production of HOCl inside cells at sites of inflammation, [18] suggest a novel HOCl-promoted free radical-mediated mechanism of cell death.

## Abbreviations:

DAPI: 4’−6-Diamidino-2-Phenylindole
DEPC: Diethylpyrocarbonate
DMPO: 5,5-Dimethyl-1-Pyrroline *N*-Oxide
ELISA: Enzyme-Linked Immune-Sorbent Analysis
ER: Endoplasmic Reticulum
ESR: Electron Spin Resonance
GAPDH: Glyceraldehydes-3-Phosphate Dehydrogenase
HRP: Horseradish Peroxidase
iNOS: Inducible Nitric Oxide Synthase
LPS: Lipopolysaccharide
MPO: Myeloperoxidase
NBS: N-Bromosuccinimide
NEM: N-Ethylmaleimide
NOX-2: NADPH Oxidase-2
SA: Succinic Anhydride
